# *Gelechiaomelkoi* sp. nov. – a new species from the Russian Altai Mountains related to the Nearctic *Gelechiamandella* Busck, 1904 (Lepidoptera, Gelechiidae), with a synopsis of *Gelechia* from the Altai Republic of Russia

**DOI:** 10.3897/zookeys.1063.71914

**Published:** 2021-10-19

**Authors:** Oleksiy Bidzilya, Peter Huemer, Jean-François Landry, Jan Šumpich

**Affiliations:** 1 Institute for Evolutionary Ecology of the National Academy of Sciences of Ukraine, 37 Academician Lebedev str., 03143, Kyiv, Ukraine Institute for Evolutionary Ecology of the National Academy of Sciences of Ukraine Kyiv Ukraine; 2 Tiroler Landesmuseen Betriebsges.m.b.H., Natural History Collections, Krajnc-Str. 1, A-6060 Hall in Tirol, Austria Tiroler Landesmuseen Betriebsges.m.b.H., Natural History Collections Innsbruck Austria; 3 Canadian National Collection of Insects, Arachnids, and Nematodes, Agriculture and Agri-Food Canada, Ottawa Research and Development Centre, C.E.F., Ottawa, Ontario K1A 0C6, Canada Canadian National Collection of Insects Ottawa Canada; 4 National Museum, Department of Entomology, Cirkusová 1740, CZ-193 00 Praha 9 – Horní Počernice, Czech Republic National Museum Praha Czech Republic

**Keywords:** Canada, distribution, DNA barcoding, Nearctic Region, new records, Palaearctic Region, Russia

## Abstract

*Gelechiaomelkoi* sp. nov. is described from the Ukok plateau and South Chuisky ridge in the Altai Mountains of Russia. The adult of the new species, including its male genitalia, is illustrated and compared with species most similar in morphology and DNA barcodes—*G.sororculella* (Hübner, 1817) and *G.jakovlevi* Krulikovsky, 1905 from the Palaearctic region, as well as *G.mandella* Busck, 1904 from Canada. This last species is redescribed based on adult specimens, including the genitalia of both sexes, and a lectotype is designated. *Gelechiasirotina* Omelko, 1986 is recorded from the Altai Republic for the first time. An updated list of six species of *Gelechia* from the Altai Mountains of Russia is given. Dorsal habitus photographs of all species are provided. The male genitalia of the lectotype of *G.jakovlevi* is illustrated for the first time.

## Introduction

During a collecting trip to the Altai Mountains in 1995, the first author collected a short series of uniformly greyish-black Gelechiidae. Despite some differences in the male genitalia and external appearance, they were identified by OB as *G.sororculella* (Hübner, 1817), and introduced under this name in a list of Lepidoptera collected on the Ukok plateau (Bidzilya et al. 2002). Six years later, seven additional males were collected in the same locality, and two males of *G.sororculella* were found in adjacent territories in the Kosh-Agach region. In 2016, two additional males were collected in Ukok by PH, and in 2017 another five males were collected at South Chuiski ridge, within the Kosh-Agach district, by JŠ. DNA barcoding of one of these specimens indicated that specimens from the Ukok plateau represented a new species (Huemer et al. 2017), with 5.94% minimum distance to the nearest Palaearctic species, *G.sororculella*, and 2.88% minimum distance to the nearest Nearctic species, *G.mandella* Busck, 1904. This last species was described from British Columbia, Canada, but it had since then not been treated in the taxonomic literature. The examination of adults during this study, particularly their genitalia, indicated its similarity to the new species from Altai as well as to *G.sororculella* and *G.jakovlevi* Krulikovsky, 1905. The new species from the Altai Mountains of Russia is described here as *G.omelkoi* sp. nov. A lectotype is designated for *G.mandella*, which is redescribed based on additional material, with male and female genitalia illustrated for the first time. Both species are compared with the Palaearctic species *G.sororculella* and *G.jakovlevi*. We also provide an updated list of *Gelechia* species known from the Altai Republic of Russia, with additional distributional information including the first record of *G.sirotina* Omelko, 1986.

## Material and methods

Specimens of the new species were collected at light as well as by sweeping during daytime or at early sunrise around shrubs of *Salix* spp. JŠ collected all specimens with portable light traps equipped with 8W ultraviolet lamps.

The studied material is deposited in the following collections:

**CBG** Centre for Biodiversity Genomics, University of Guelph, Ontario, Canada

**CNC** Canadian National Collection of Insects, Arachnids, and Nematodes, Ottawa, Canada

**NMPC** National Museum, Prague, Czech Republic

**TLMF** Tiroler Landesmuseum Ferdinandeum, Innsbruck, Austria

**USNM** United States National Museum,Washington, D.C., USA

**ZIN** Zoological Institute, Russian Academy of Sciences, Saint-Petersburg, Russia

**ZMKU** Zoological Museum, Kyiv National Taras Shevchenko University, Kyiv, Ukraine

Male and female genitalia were dissected and prepared using standard methods for Gelechiidae (Landry 2007; Huemer and Karsholt 2010). Slide-mounted genitalia were prepared and photographed as described by Landry et al. (2013) and Bidzilya et al. (2020).

The descriptive terminology largely follows Huemer and Karsholt (1999), except cucullus instead of valva and phallus instead of aedeagus.

### DNA Barcoding

A tissue sample from a specimen of *Gelechiaomelkoi* sp. nov. was successfully processed at the Canadian Centre for DNA Barcoding (CBG, Biodiversity Institute of Ontario, University of Guelph) (deWaard et al. 2008), resulting in a 658 base-pair full DNA barcode segment of the mitochondrial COI gene (cytochrome c oxidase 1). Complementary public sequences of *G.mandella* (n=13), *G.sororculella* (n=17) and *G.rhombella* (n=10) from BOLD systems v. 4.0. (http://www.boldsystems.org; Ratnasingham and Hebert 2007) were used for analysis (Table 1). Degrees of intra- and interspecific variation of DNA barcode fragments were calculated under the Kimura 2-parameter model of nucleotide substitution using the analytical tools of BOLD. A neighbor-joining tree of DNA barcode data of selected taxa (Table 1) was constructed using MEGA 6 (Tamura et al. 2013) under the Kimura 2 parameter model for nucleotide substitutions.

**Table 1.** Analysed specimens of *Gelechia* spp. from BOLD

Species	Sample ID	Process ID	GenBank	BIN
*Gelechiamandella*	08BBLEP-02943	LPAB601-08	KM542545	BOLD:AAG0039
	08BBLEP-02962	LPAB620-08	KM549242	BOLD:AAG0039
	08BBLEP-03043	LPAB701-08	KM542418	BOLD:AAG0039
	BIOUG22954-B10	GMOCL291-15	MG358112	BOLD:AAG0039
	BIOUG23126-F09	GMOLH046-15	MG360795	BOLD:AAG0039
	BIOUG23265-E04	GMOLH161-15	MG363316	BOLD:AAG0039
	BIOUG44827-B07	GMOLF029-19		BOLD:AAG0039
	BIOUG44827-B08	GMOLF030-19		BOLD:AAG0039
	BIOUG44827-B10	GMOLF032-19		BOLD:AAG0039
	BIOUG44832-B12	GMORG046-19		BOLD:AAG0039
	CNCLEP00067704	MNAJ551-09		BOLD:AAG0039
	CNCLEP00067705	MNAJ552-09		BOLD:AAG0039
	CNCLEP00100431	CNCLA1217-13		BOLD:AAG0039
*Gelechiarhombella*	MM02568	LEFIB736-10	HM871614	BOLD:AAE6372
	MM09529	LEFIE614-10	HM874337	BOLD:AAE6372
	MM03481	LEFIC137-10	HM871983	BOLD:AAE6372
	TLMF Lep 15352	ABOLA330-14	MN805653	BOLD:AAE6372
	TLMF Lep 15357	ABOLA335-14	MN805882	BOLD:AAE6372
	TLMF Lep 16781	ABOLA821-15	MN803821	BOLD:AAE6372
	TLMF Lep 24269	LEAST911-17	MN805984	BOLD:AAE6372
	KLM Lep 08814	LEAST1479-18	MN803550	BOLD:AAE6372
	KLM Lep 12426	LEAST1671-18	MN806057	BOLD:AAE6372
	MM05043	LEFIJ14972-20		BOLD:AAE6372
*Gelechiasororculella*	MM13873	LEFIA945-10	HM387078	BOLD:AAC8633
	MM00668	LEFIB214-10	HM871118	BOLD:AAC8633
	MM00669	LEFIB215-10	HM871119	BOLD:AAC8633
	MM09008	LEFIE419-10	HM874143	BOLD:AAC8633
	TLMF Lep 03819	PHLAD644-11	JN271047	BOLD:AAC8633
	TLMF Lep 05290	PHLAF120-11	MN804563	BOLD:AAC8633
	TLMF Lep 07445	PHLAG766-12	MN806665	BOLD:AAC8633
	TLMF Lep 08880	PHLAI385-13	MN804332	BOLD:AAC8633
	TLMF Lep 09231	PHLAI669-13	MN806319	BOLD:AAC8633
	TLMF Lep 12390	LEATC408-13	MN804176	BOLD:AAC8633
	TLMF Lep 11904	LEATE492-13	MN803907	BOLD:AAC8633
	TLMF Lep 16768	ABOLA808-15	MN803611	BOLD:AAC8633
	TLMF Lep 17098	ABOLB093-15	MN806268	BOLD:AAC8633
	TLMF Lep 21377	LEKOB014-16	MN804141	BOLD:AAC8633
	KLM Lep 12406	LEAST1651-18	MN806454	BOLD:AAC8633
	KLM Lep 14931	LEASV1480-19		BOLD:AAC8633
	KLM Lep 14936	LEASV1485-19		BOLD:AAC8633
*Gelechiaomelkoi*	TLMF Lep 20453	LEALT230-16		BOLD:ADD9926

## Results

### Gelechia omelkoi sp. nov.

Taxon classificationAnimaliaLepidopteraGelechiidae

F2881F8E-6D85-5E15-9CB8-353E9FF067BD

http://zoobank.org/831C091D-D8DD-44C0-A3E5-9732381EBA22

Figures 2 , 6 , 7 , 14 , 15–16

#### Material examined.

Holotype [Russia] • ♂; Altai, Kosh-Agatch distr., Ukok plateau; 2200 m; 19 Jul 2001; Bidzilya leg.; ZMKU.

#### Paratypes.

Russia • 6 ♂; same collection data as for holotype; 1, 10, 20, 24, 25 Jul 2001; [genitalia slide number] 286/20, O. Bidzilya • 3 ♂; same collection data as for holotype; 22 Jul 1995 [genitalia slide number] 62/03, O. Bidzilya, all ZMKU• 2 ♂; Altai Republic, Kosh-Agatch distr., Northern part of Ukok plateau, Zhumaly river basin; 2400–2500 m; 4–6 Aug 2016; P. Huemer and B. Wiesmair leg. [Barcode identification number] TLMF Lep 20453; TLMF • 4 ♂♂; Altai, Belyashi [Dzhazator] env. (25 km NW), confluence of Argut and Karagem rivers; 49.865°N, 87.173°E; 1400 m; rocky steppe; 27–28 Jul 2017 [genitalia slide number] 21257, J. Šumpich; J. Šumpich leg. • 1 ♂; Altai, Belyashi (Dzhazator) env. (56 km SE), Dzhazator valley, 49.63°N, 88.20°E, mountain meadows near Tara river; 2300 m; 25–26 Jul 2017; [genitalia slide number] 21261, J. Šumpich; J. Šumpich leg.; all NMPC.

**Diagnosis.** The new species differs externally from most other Palaearctic species of *Gelechia* by the uniformly blackish-grey forewing without markings. *Gelechiamandella* and *G.sororculella* are similarly dark but without glossy forewings and with at least some indication of paler markings. The male genitalia are similar to those of *G.mandella*, *G.sororculella* and *G.jakovlevi*. The differences among these taxa are summarized in Table 2.

**Table 2.** Characters separating *G.omelkoi* sp. nov., *G.mandella*, *G.sororculella* and *G.jakovlevi*.

Characters	*omelkoi*	*mandella*	*sororculella*	*jakovlevi*
Apex of phallus	Short, weakly pointed	Elongate, pointed, broad at base	Elongate, pointed, narrow at base	Elongate, pointed, narrow at base
Ratio middle part of phallus /caecum	0,5	0,7	0,7	0,5
Fultura superior	Weakly divided, not extended to anteromedial emargination of tegumen	Weakly divided, not extended to anteromedial emargination of tegumen	Weakly divided, not extended to anteromedial emargination of tegumen	Deeply divided, extended to anteromedial emargination of tegumen
Sacculus	3/4–4/5 length of cucullus	2/3–3/4 length of cucullus	4/5 length of cucullus	4/5 length of cucullus
Posterior margin of uncus	Straight	Straight	Straight	Weakly emarginate

#### Description.

Adult (Figs 2, 15, 16). Forewing length 6.5–7.2 mm (mean = 6.7, n=10). Wingspan 13.8–15.0 mm. (mean = 14.4, n=10). Head, thorax and tegulae black, with rare grey-tipped scales on frons, labial palpus black mixed with white, underside of palpomere 2 with brush of long scales separated by medial gap, white on the inner side, scape black, flagellomeres black, ringed with grey, densely ciliated beneath, forewing overall matt, covered with grey brown- or grey-tipped scales, without markings, fringe grey, brown-tipped; hindwing grey, veins mottled with brown.

In male, sternum VIII rounded, anterior part narrow, reverse-trapezoid; tergum VIII elongate, tongue-shaped, with paired long coremata (Fig. 14).

Male genitalia (Figs 6, 7). Uncus broadly rounded, two times broader than long, posterior margin weakly serrated, edged with long setae, distal sclerite of gnathos absent, lateral sclerites slender, short, culcitula broad, pillow-shaped, fultura superior extended anteriorly to about 2/3 length of tegumen, not reaching anteromedial emargination of tegumen, tegumen nearly parallel-sided, 2.5 times longer than broad at base; cucullus slender, of even width, extended to apex of uncus, sacculus in its broadest part 2–3 times as broad as cucullus, apex tapered, curved inwards, extended to 3/4–4/5 length of cucullus, vinculum broad, medial processes rounded, broadly separated; saccus tapered, extended far beyond apex of pedunculi; phallus slightly shorter than tegumen, medial section nearly parallel-sided, caecum distinctly inflated, about 2 times as broad as phallus, apex short, weakly pointed, lateral lobe reverse V-shaped, lateral process short, thorn-shaped, medial sclerite slender, elongate; bulbus ejaculatorius moderately long, sack-shaped, with small irregularly shaped lamina.

Female genitalia. Unknown.

#### Biology.

Part of the type series, including the holotype, was collected by netting during early sunrise around dwarf willows (*Salixglauca* and others) at altitudes from 2200 to 2500 m. It is highly likely that one of these *Salix* species is a host plant for the larvae, and that the new species is restricted in its distribution to mountain areas where its possible host plant occurs. Other specimens were attracted to light in the same habitats, in mountains meadow or rocky steppe from 1400 to 2500 m (Figs 23, 24). *Gelechiasororculella* is also known from neighboring territories of Altai, but was observed in river valleys (Chuya, Chagan). This species is associated with several species of *Salix* (Huemer and Karsholt 1999), but not with the dwarf willows presumed to be the host for *G.omelkoi* sp. nov.

**Figure 1.**
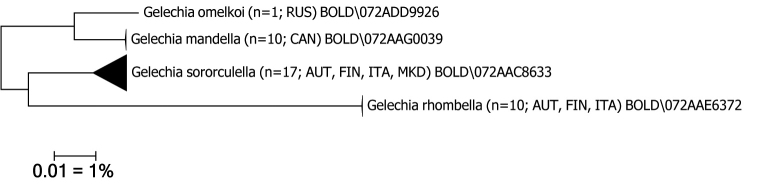
Neighbor-Joining tree of *Gelechiaomelkoi* sp. nov. and nearest European and North American *Gelechia* spp. in BOLD, with the generic type species *Gelechiarhombella* as outgroup (Kimura 2-parameter, constructed with MEGA 6 cf. Tamura et al. 2013), only sequences >500 bp considered. The scale bar only applies to internal branches between species. Width of triangles represents sample size, depth represents genetic variation within the cluster. Source: DNA Barcode data from BOLD (Barcode of Life Database, cf. Ratnasingham and Hebert 2007).

Molecular data. BIN: BOLD:ADD9926 (n=1). The minimum distance to the nearest neighbour, the North American *G.mandella*, is 2.88%, whereas it is 5.94% distant from the nearest Palaearctic *G.sororculella* (Fig. 1).

#### Distribution.

Russia: Altai Republic, Ukok plateau and South Chuisky ridge.

#### Etymology.

The new species is named in honour of Mikhail M. Omelko (Federal Scientific Center of East Asia Terrestrial Biodiversity, Far Eastern Branch, Russian Academy of Sciences, Vladivostok, Russia), in recognition of his contribution to the study of Gelechiidae, and the genus *Gelechia* in particular. The species name is a noun in the genitive case.

### Gelechia mandella

Taxon classificationAnimaliaLepidopteraGelechiidae

Busck, 1904

3BA105D5-1E0C-5759-96CB-43922CA5E2E7

Figs 3–5 , 8 , 9 , 12 , 13

Gelechia mandella Busck, 1904. – Proceedings of the United States National Museum 27 (1375): 759. Type locality: Kaslo, British Colombia, Canada.

#### Material examined.

[Canada] • 16 ♂; Alberta, Nordegg, [54.470°N, 116.075°W], various dates from 29 Jun – 6 Aug 1921; [barcoded male 4 Jul 1921]; bred from larva on Willow; J. McDunnough leg. [specimen number] CNCLEP00100431; [genitalia slide number] MIC 8484; [other males numbers] CNCLEP00100430–100433, CNCLEP00127961–127973 • 1 ♀; same collection data as for proceeding, 10 Jul 1921 [specimen number] CNCLEP00127968; [genitalia slide number] MIC 8485 • 2 ♂; Yukon, km 140.5 Dempster Hwy, [65.069°N, 138.129°W], 900 m, 28 Jul 1980; D. Wood and J. Lafontaine leg.; [specimen number] CNCLEP00067704–67705; genitalia slide number [MIC 8486]; all in CNC.

**Figures 2–5.**
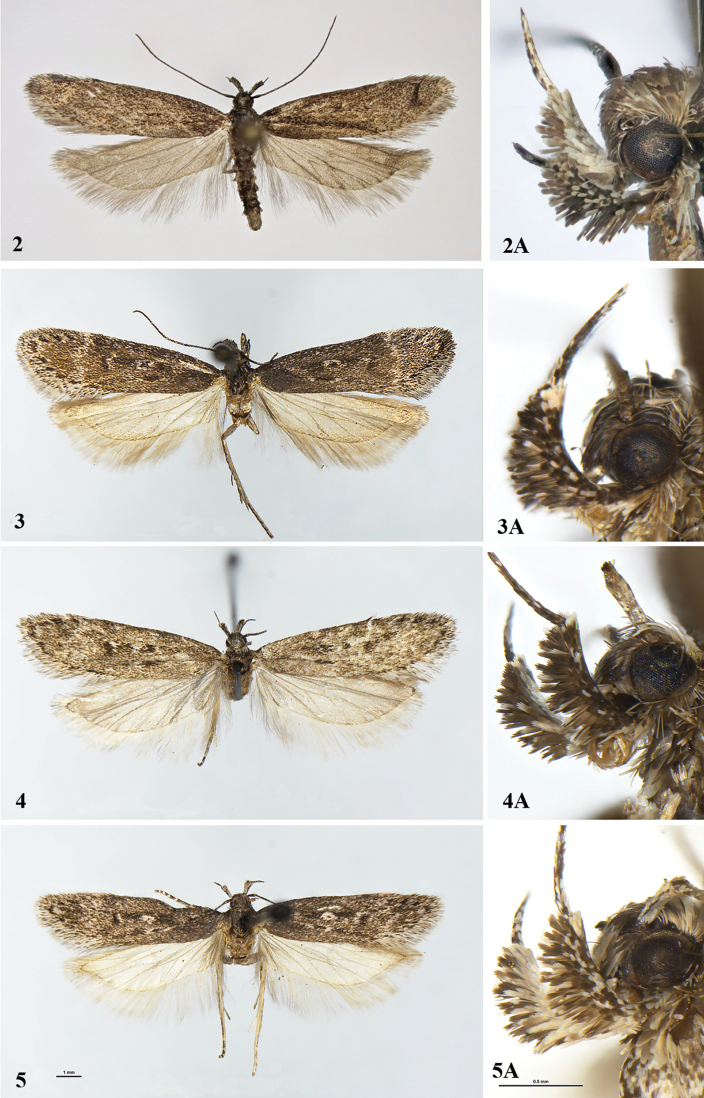
*Gelechia* spp. Adults. **2, 2A***Gelechiaomelkoi* sp. nov. **2** adult, holotype **2A** head, paratype **3–5***G.mandella***3, 3A** male, Alberta **3** adult **3A** head **4, 4A** male, Yukon **4** adult **4A** head **5, 5A** female Alberta **5** adult **5A** head.

**Figures 6–11.**
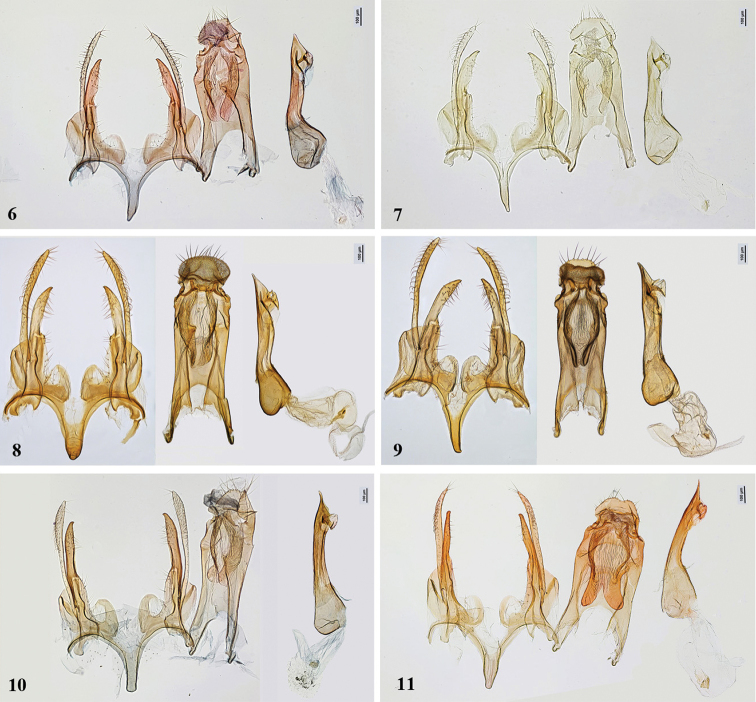
*Gelechia* spp., male genitalia. **6, 7***G.omelkoi* sp. nov., paratypes **6** genitalia slide 286/20, O. Bidzilya **7** genitalia slide 62/03, O. Bidzilya **8, 9***G.mandella***8** Alberta, genitalia slide MIC 8484 **9** Yukon, genitalia slide MIC 8486 **10***G.sororculella*, genitalia slide 287/20, O. Bidzilya **11***G.jakovlevi*, lectotype, genitalia slide 309/20, O. Bidzilya.

#### Diagnosis.

*Gelechiamandella* is a blackish-grey, medium-sized species with a black streak interrupted by diffuse white spots in the middle of the forewing, a black streak in fold and a diffuse white subapical fascia. The wing pattern resembles that of the Holarctic species *Gelechiasabinellus* (Zeller, 1839), but it is darker and predominantly black rather than grey. Additionally, *G.sabinellus* has strikingly differently coloured scales on the labial palps. The Palaearctic *G.sororculella* looks nearly indistinguishable externally (Fig. 19).

#### Redescription.

Adult (Figs 3–5). Forewing length 7.8–9.4 mm (mean = 8.6, n=18). Wingspan 15.9–18.7 mm (mean = 17.1, n=16). Head, thorax and tegulae greyish black, labial palpus black mixed with white, palpomere 2 underside with brush of long scales divided by medial gap, inner side entirely white in some specimens, scape black, flagellomeres black, ringed with light grey, forewing greyish black, sparsely mixed with white-tipped scales, fold with black medial streak edged with white from both ends, indistinct black streak in middle 2/3 interrupted by large white spot at 1/2 and much smaller white spot at 2/3, diffuse white fascia at about 3/4, termen black-spotted, cilia white, black-tipped; hindwing light grey with grey cilia and distinctly darkened veins.

**Figures 12–14.**
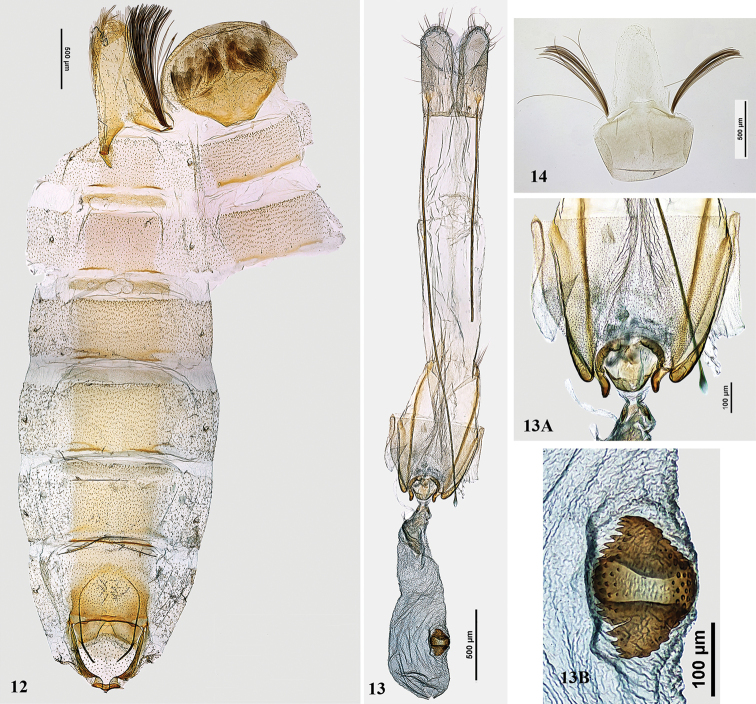
*Gelechia* spp., genitalia and abdomen. **12, 13***G.mandella*. **12** abdomen, male, genitalia slide MIC 8484 **13A, B** Female genitalia, slide MIC 8485 13A segment VIII (enlarged) **13B** Signum (enlarged) **14***G.omelkoi* sp. nov., male segment VIII, genitalia. slide 62/03, O. Bidzilya.

In male, sternum VIII rounded in distal part, reverse trapezoid basally; tergum VIII elongate, tongue-shaped, with paired, long coremata (Fig. 12).

Male genitalia (Figs 8, 9). Uncus broadly rounded, twice wider than long, posterior margin weakly serrated, edged with long setae, distal sclerite of gnathos absent, lateral sclerites slender, short, culcitula slightly wider than uncus, pillow-shaped, fultura superior extended anteriorly to about 2/3 length of tegumen, not reaching anteromedial emargination of tegumen, tegumen nearly parallel-sided, 2.5 times as long as broad at base; cucullus slender, of even width, extended to apex of uncus, sacculus in its broadest part 1.5–2.0 times as broad as cucullus, apex tapered, curved inwards, extended to 2/3–3/4 length of cucullus, vinculum broad, medial processes rounded, broadly separated; saccus weakly or distinctly narrowed apically, extended far beyond apex of pedunculi; phallus slightly shorter than tegumen, medial portion nearly straight or with dorsal side slightly curved, caecum weakly inflated, about 1.5 times as broad as phallus, apex moderately elongate, triangularly pointed with comparatively broad base, dorsal lobe beak-shaped and recurved, lateral process short, thorn-shaped, medial sclerite slender, elongate; bulbus ejaculatorius elongate, sack-shaped, with small, irregularly shaped lamina.

Female genitalia (Figs 13, 13A, 13B). Papillae anales elongate, subovate, with straight anterior margin; apophyses posteriores three times as long as segment VIII, apophyses anteriores reduced to melanized bands fused to lateral wall of sternum VIII; sternum VIII three times longer than broad, with narrow, sclerotized lateral rods, wrinkled along medial membranous zone, with strongly sclerotized short anterolateral drop-shaped processes confluent with apices of apophyses anteriores; subgenital plate small, band-shaped, with short, pointed anterior protrusions near anterior margin of sternum VIII; ostium rounded, with distinct posterolateral edging connected anteriorly with base of apophyses anteriores; antrum cylindrical, colliculum short, trapezoid, laterally sclerotized; ductus bursae very short, broadened into corpus bursae, with indistinct transition, corpus bursae as long as and slightly wider than adjacent part of ductus bursae, signum plate subovate, with serrate margins and broad transverse medial groove.

#### Biology.

Adults have been collected from late June to early August in Alberta and in late July in Yukon. Two specimens from Nordegg, Alberta were reared from an unspecified willow.

Molecular data. BIN: BOLD:AAG0039. The intraspecific average distance of the barcode region is 0.14% (n=13, data from BOLD). The minimum distance to the nearest neighbour, the Palaearctic *G.omelkoi* sp. nov., is 2.88% (Fig. 1).

#### Distribution.

Canada: British Columbia, Alberta, Yukon (new record), Northwest Territories (new record). Two alleged records from Montana, USA in SCAN (2021) are represented by photographs taken on 14–15 May 2018 near the town of Missoula in the mountainous western part of the state. Although the superficial appearance of the moths on the photos makes it possible that this be *G.mandella*, their identity remains unverified. The same website also shows two Northwest Territories records, which are actually sourced from two BOLD public records analyzed here (BIOUG23265-E04 and BIOUG23126-F09 deposited in CBG; see Table 1). The record from Quebec in Pohl et al. (2018) was based on a female specimen in CNC from Forestville (specimen # CNCLEP00100429), which has since been barcoded and belongs to a different BIN (BOLD:AAH6283). It is here excluded and likely represents a different species.

#### Remarks.

Busck (1904) described *Gelechiamandella* from an unspecified number of specimens, as indicated by a size range accompanying the original description. There is a series of specimens of *G.mandella* identified by Busck in the collection of USNM. We assume four of them, with red type labels, are from Busck’s original series. None of these syntypes has a locality label, only a Dyar field number, which corresponds with Kaslo, British Columbia (Canada). This series comprises two females collected 15.08.1903 (USNM slide #6773 (genitalia), USNMENT01480487 and USNM slide #6779 (wings), USNMENT01480485); one specimen without an abdomen, collected 13.08.1903 (USNMENT01480486); and one dissected male collected 5.08.1903. This last specimen, labelled “type No. 7859, U.S.N.M”, “Genitalia Slide 6775, by AB, ♂, USNM”, “*Gelechiamandella* Busck, Type” (USNMENT00835335) was incorrectly published as the “holotype” by Brown et al. (2004). Photographs of the specimen, its labels, and the genitalia are available online (https://collections.nmnh.si.edu/search/ento/?ark=ark:/65665/38eb1f15df800489fac64727ff945379c). At one time, the USNMENT00835335 specimen was labelled with “Mesilla, NM [New Mexico].” This was likely due to a mix-up when labels were removed from the pins to be photographed and the Mesilla label belongs to another type, possibly *Gelechiamalindella* Busck, 1910 [a junior synonym of *Friseriacockerelli* (Busck, 1903)]. The label error for the USNMENT00835335 specimen is now corrected. Here, we designate the USNMENT00835335 specimen among the likely syntypes as lectotype of *G.mandella*, to stabilize nomenclature.

The CNC series from Nordegg, Alberta collected in 1921 was identified as *G.mandella* by Annette Braun. Despite the difficulty to interpret some characters of the male genitalia from the photo of the lectotype slide of *G.mandella*, visible features match those of the barcoded specimens. Taking also into consideration the similarity in external appearance, we are confident that specimens from Yukon and Alberta represent *G.mandella*.

## Discussion

The genus *Gelechia* is represented by 22 species in Europe, and the European fauna was revised and studied in detail by Huemer and Karsholt (1999). In North America, 40 valid species are recognized, but the genus has never been the object of any revision and several names remain of uncertain identity (Lee et al. 2009). In Russia, the genus was revised for the Far East (Omelko 1986), and the data on the distribution of 24 species throughout the country were summarized (Ponomarenko 2019). Until recently, 10 species were recorded from Siberia, including the rather unexpected finding of *Gelechiarepetitrix* Meyrick, 1931 from the Omsk region (Ponomarenko and Knyazev 2020). Currently, six species of *Gelechia* are known from the Altai, but records of additional species (e.g., *G.turpella* ([Denis & Schiffermüller], 1775) are expected. Below, we provide a list of *Gelechia* species known from the Altai Republic of Russia, with updated information on their distribution and corresponding references.

### Gelechia sororculella

Taxon classificationAnimaliaLepidopteraGelechiidae

(Hübner, [1817])

0C1F38C4-7F84-524B-AA21-94EB715DAA99

Figs 10 , 19

#### Records.

Bidzilya et al. 2002: 207. Misidentification of G.omelkoi sp. nov.

#### Material examined.

Russia • 1 ♂; Altai Republic, Shebalino distr., Cherga env.; 17 Jul 1995; P. Ustjuzhanin leg.; ZMKU • 1 ♂, Russia, Altai, Kosh-Agatch distr., 15 km from Beltir. vil. up on Tchagan river; steppe; 2200 m; 13 Aug 2000; O. Bidzilya leg.; [genitalia slide number] 287/20, O. Bidzilya; ZMKU • 1 ♂; Altai, Kosh-Agatch env., Tchuja river bank; on trunk of Salix sp.; 17 Aug 2000; O. Bidzilya leg.; ZMKU.

Kyrgyzstan • 1 ♂; 5 km S of At-Bashi, Narynskaya oblast; 15 Aug 1981; S. Sinev leg.; ZIN.

#### Remarks.

The previous record of this species from Ukok plateau in Altai (Bidzilya et al. 2002: 207) refers to *G.omelkoi* sp. nov.

#### Distribution.

Palaearctic Region from Spain to Russian Far East (Huemer and Karsholt 1999; Ponomarenko 2019); Kyrgyzstan (new record).

##### *Gelechiaomelkoi* sp. nov.

**Material examined.** (see above).

**Distribution.** Russia (Altai Mts).

### Gelechia jakovlevi

Taxon classificationAnimaliaLepidopteraGelechiidae

Krulikovsky, 1905

C07D2A50-FE68-5C03-A806-14599B4C1AC5

Figs 11 , 17 , 18

#### Records.

Bidzilya 2002: 69.

#### Material examined.

Russia • 1 ♀; Altai, Ongudai distr., 15 km from Iodro vil. down on Tchuja river; 6 Aug 2000; O. Bidzilya leg.; [genitalia slide number] 293/20, O. Bidzilya; ZMKU • 1 ♂, Russia, Altai, Belyashi (Dzhazator) env. (25 km NW), confluence of Argut and Karagem rivers; rocky steppe; 49.865°N, 87.173°E; 1400 m; 27–28 Jul 2017 [genitalia slide number] 21265, J. Šumpich; J. Šumpich leg.; NMPC.

#### Distribution.

Northern and eastern Europe, Russia: European part, Tomsk region, Altai, Buryatia (Huemer and Karsholt 1999; Ponomarenko 2019); Mongolia (ssp. mongoliae Emeljanov & Piskunov, 1982).

#### Remarks.

*Gelechiajakovlevimongoliae* was described based on a female from Songino, western Mongolia. The status of this taxon needs clarification after examination of a male, which is unknown to us.

### Gelechia muscosella

Taxon classificationAnimaliaLepidopteraGelechiidae

Zeller, 1839

B9B7AB7F-B2CC-509D-AF83-26C4520F465B

Fig. 20

#### Records.

Bidzilya 2002: 68.

#### Material examined.

Russia • 1 ♂; Gornoaltaisk; 15 Jul 1997; A. Lvovsky leg.; ZIN • 1 ♂; Altai, Aktash vill.; grassy steppe, rocks; 50.320°N, 87.60°E; 1400 m; 11 Jul 2014; J. Šumpich leg.; NMPC.

**Figures 15–22.**
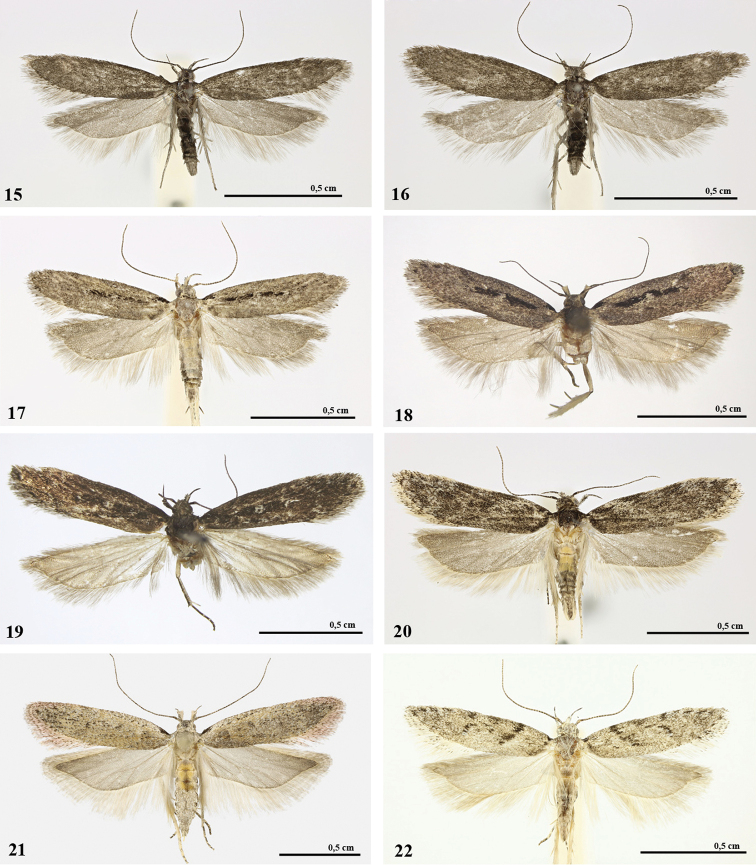
*Gelechia* spp., adults. **15, 16***G.omelkoi* sp. nov., paratypes, males. **15** Dzhazator. **16** Karagem. **17, 18***G.jakovlevi*. **17** Male, Karagem. **18** Female, Iodro **19***G.sororculella*, male, Cherga **20***G.muscosella*, male, Aktash **21***G.hippophaella*, female, Karagem **22***G.sirotina*, male, Karagem.

#### Distribution.

Palaearctic Region from Great Britain to Far East of Russia and China: Qinghai, Gansu, Shaanxi (Huemer and Karsholt 1999; Li 2002).

**Figures 23, 24.**
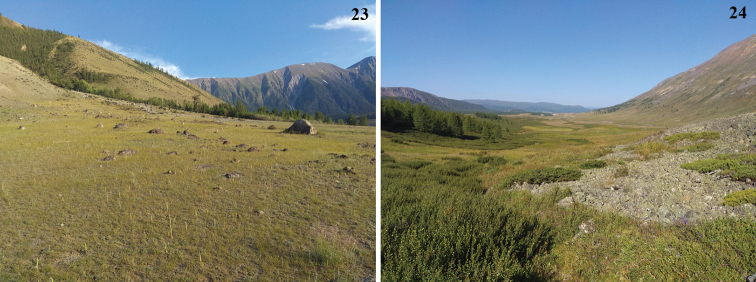
Habitats of *Gelechiaomelkoi* sp. nov. **23** Steppe near the confluence of Argut and Karagem rivers **24** mountain steppe near Dzhazator (photographs by Jan Šumpich).

### Gelechia hippophaella

Taxon classificationAnimaliaLepidopteraGelechiidae

(Schrank, 1802)

998820CF-6F79-5AD9-B691-EC23F109DA4E

Fig. 21

#### Records.

Piskunov 1981: 669.

#### Material examined.

Russia • 1 ♀; Altai, Belyashi (Dzhazator) env. (25 km NW), confluence of Argut and Karagem rivers; rocky steppe; 49.865°N, 87.173°E; 1400 m; 27–28 Jul 2017 J. Šumpich leg.; NMPC.

#### Distribution.

Northern, central and south-eastern Europe; Siberia: Altai, Tuva, Buryatia; China: Ningxia (Huemer and Karsholt 1999; Li 2002; Ponomarenko 2019), unconfirmed record from Mongolia (Piskunov 1990).

### Gelechia sirotina

Taxon classificationAnimaliaLepidopteraGelechiidae

Omelko, 1986

BE2F110C-0B03-518D-B018-E38484E241B2

Fig. 22

#### Material examined.

Russia • 1 ♂; Altai, Belyashi (Dzhazator) env. (25 km NW), confluence of Argut and Karagem rivers; rocky steppe; 49.865°N, 87.173°E; 1400 m; 27–28 Jul 2017 [genitalia slide number] 19922, J. Šumpich; J. Šumpich leg.; NMPC.

#### Distribution.

Belarus; Tajikistan (Piskunov 1989); Russia: Altai (new record), Tuva, Zabaikalskiy krai, Primorskiy krai (Ponomarenko 2019).

## Supplementary Material

XML Treatment for Gelechia omelkoi

XML Treatment for Gelechia mandella

XML Treatment for Gelechia sororculella

XML Treatment for Gelechia jakovlevi

XML Treatment for Gelechia muscosella

XML Treatment for Gelechia hippophaella

XML Treatment for Gelechia sirotina
